# Supplementary material for: Unprecedented switching endurance affords for high-resolution surface temperature mapping using a spin-crossover film
Source: Nat Commun. 2020 Jul 17;11:3611. doi: 10.1038/s41467-020-17362-7 (PMC7367879; doi:10.1038/s41467-020-17362-7)
Supplement: Supplementary file 1 — Supplementary Information [file 41467_2020_17362_MOESM1_ESM.pdf]

Supplementary information

Unprecedented switching endurance affords for high-resolution surface temperature mapping using a spin-crossover film

Karl Ridier[1], Alin-Ciprian Bas[1], Yuteng Zhang[1], Lucie Routaboul[1], Lionel Salmon[1], Gábor Molnár[1*], Christian Bergaud[2] & Azzedine Bousseksou[1*]

[1]Laboratoire de Chimie de Coordination, CNRS UPR 8241, 205 route de Narbonne, F–31077 Toulouse, France.

[2]Laboratoire d'Analyse et d'Architecture des Systèmes, CNRS UPR 8001, 7 avenue du Colonel Roche, F–31400 Toulouse, France.

Supplementary Figures

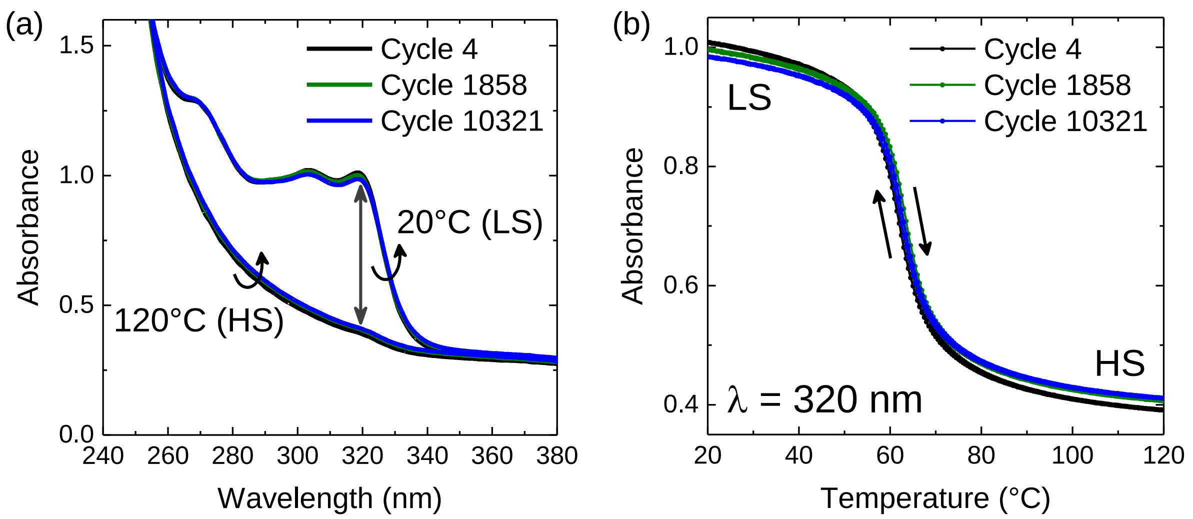

Supplementary Figure 1. Optical absorbance measurements on thin films of 1. (a) Optical absorbance spectra acquired at 20 °C (LS state) and 120 °C (HS state) on a 200-nm-thick film of **1** deposited on top of a fused silica substrate after 4, 1858 and 10321 thermal cycles in air. The films of **1** exhibit intense absorption bands in the UV range (260–340 nm) at room temperature (LS state), which are completely bleached in the HS state. **(b)** Thermal evolution of the optical absorbance at $\lambda = 320$ nm after the successive thermal cycles. Although a slight decrease in the amplitude of the absorbance change (-7 %) is observed (comparable with the experimental uncertainties), these measurements demonstrate the excellent stability and the strong robustness of the spin-transition properties over the two-week-long experiment, the switching temperature remaining invariant after more than 10,000 consecutive thermal cycles in air.

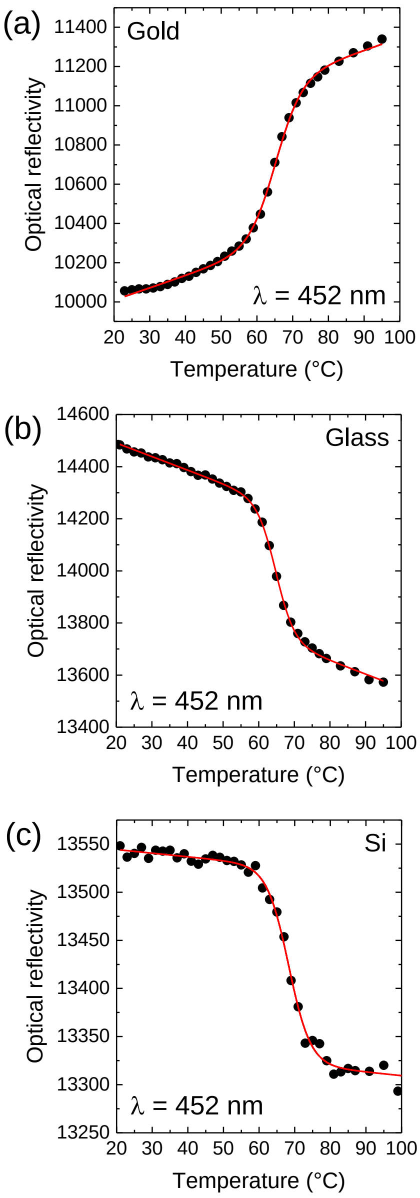

Supplementary Figure 2. Optical reflectivity properties of thin films of 1. Typical thermal evolution of the optical reflectivity (λ = 452 nm) of a 200-nm-thick film of **1** deposited on a **(a)** gold, **(b)** glass and **(c)** silicon surface. The relative variation of the optical reflectivity ($\Delta R/R$) is *ca.* +7 %, -4 % and -1.5 %, respectively, due to the switching from the LS to the HS state. These sizeable variations of the optical reflectivity upon the spin transition are primarily related to the significant volume expansion of the crystal lattice ($\Delta V/V$ = 4.5 % for **1**)[1] and the concomitant refractive index variation, which accompanies the spin-state switching.

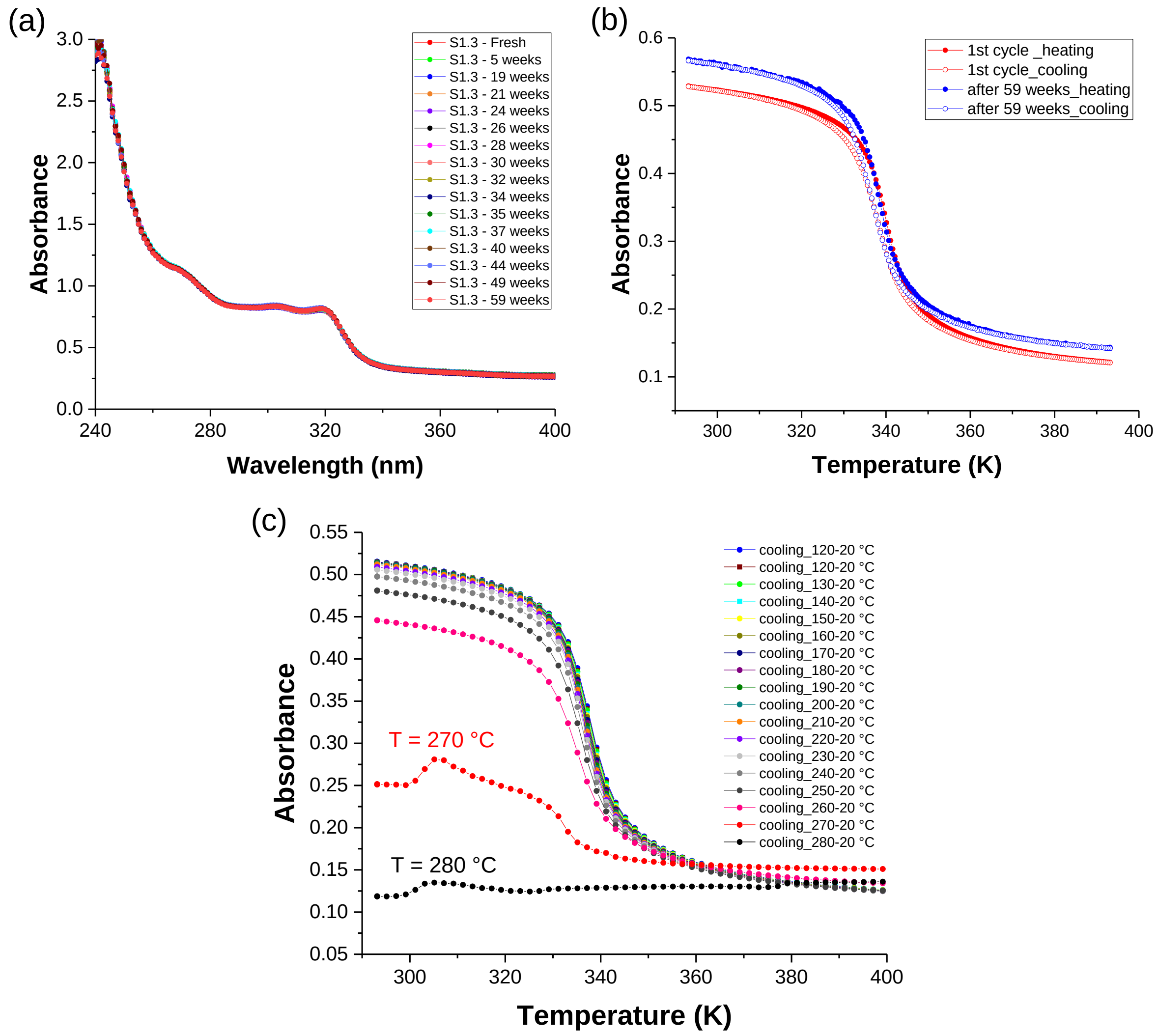

Supplementary Figure 3. Stability of the films of 1 upon storage in ambient air and high-temperature treatment. (a) Absorbance spectra of a 175-nm-thick film of **1** recorded regularly at room temperature along a period of 59 weeks of storage in ambient air. These measurements demonstrate the invariance of the LS absorption bands over this period of 59 weeks. **(b)** Thermal spin-transition curves, deduced from temperature-dependent UV absorbance measurements (λ = 320 nm) of the film freshly prepared, and after 59 weeks of storage in air. Remarkably, the switching temperature, the shape of the transition curve and the completeness of the SCO remain virtually unaltered (within the experimental accuracy) over this period of more than one year. The vertical upshift of the transition curve may be attributed either to an increased light scattering of the films (*e.g.* because of dust) and/or to slight differences in the experimental conditions (*e.g.* position of the cryostat *vs.* the light beam of the spectrophotometer, *etc.*). **(c)** Thermal spin-transition curves recorded at a scan rate of 4 °C.min^{-1} for a 175-nm-thick film of **1**. For each thermal cycle, the heating range was extended by a step of 10 °C. The spin transition is found to be reproducible up to 230 °C. (N.B. Above this temperature, the molecules start to sublimate.)

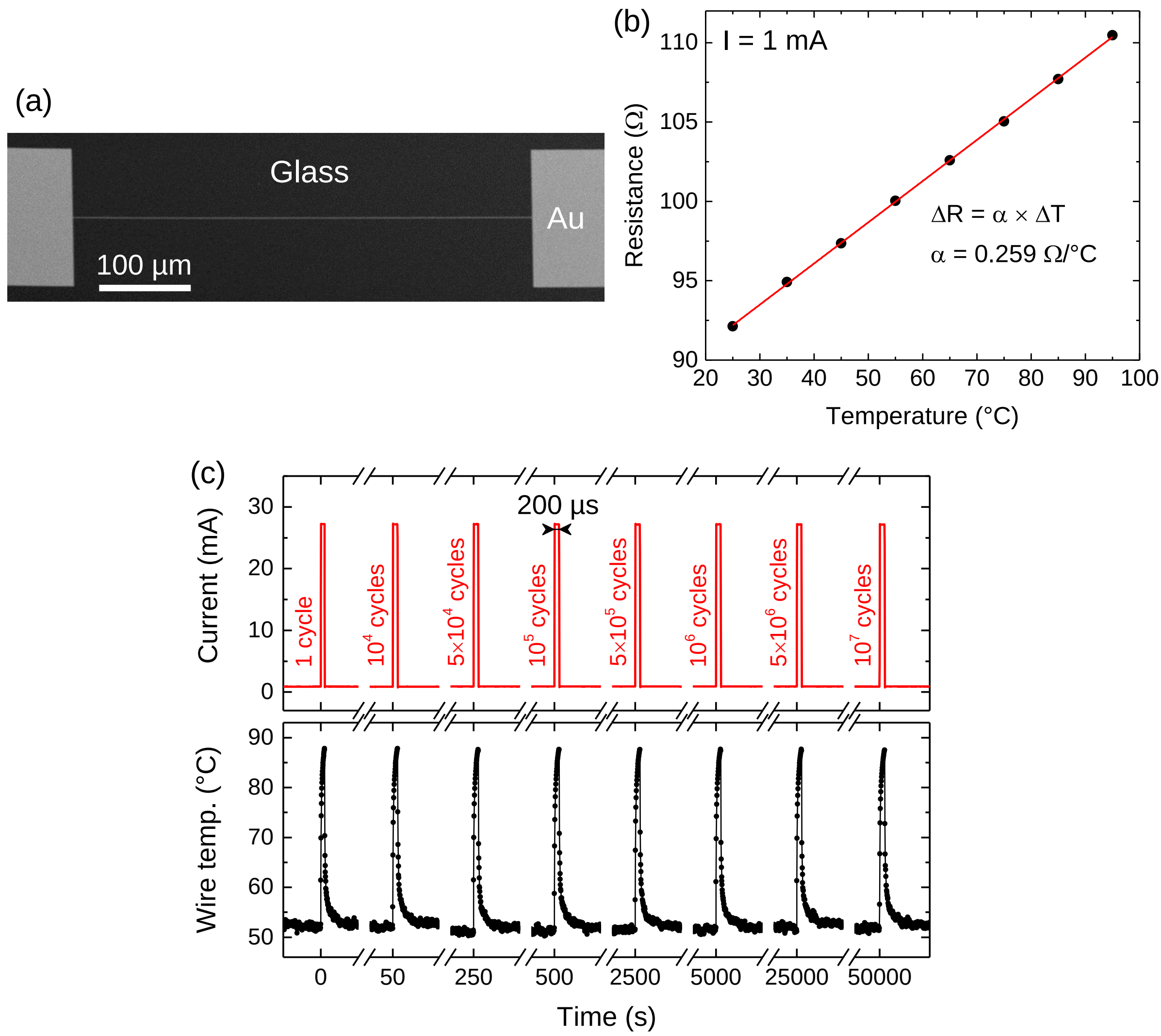

Supplementary Figure 4. Switching endurance of a thin film of 1 on a Joule-heated microwire. (a) Scanning electron microscopy (SEM) image of a 2-mm-long, 2-µm-wide, 300-nm-thick gold wire on top of a glass substrate, used as a fast micro-heater. **(b)** Variation of the electrical resistance of the wire, measured at low-bias current (1 mA), as a function of the base temperature (T_b) of the substrate, controlled with a variable-temperature stage. The linear temperature coefficient of the wire is found to be α = 0.259 Ω.°C^{-1}. **(c)** Time evolution of the applied electrical current (top panel) and the wire temperature (bottom panel) at selected times of the thermal-cycling experiment. In order to expose the film of **1**, deposited on top of the wire, to a large number of thermal cycles (10^7) within a reasonable time (< 14 h), the base temperature of the substrate was fixed at T_b = 50 °C and 200-µs-long current pulses (28 mA) were applied to the wire (R_{wire} = 98.7 Ω) at a frequency of 200 Hz. In this way, the temperature of the wire was modulated between 50 °C and 88 °C. The wire temperature was monitored in real time using a differential resistance measurement setup[2,3], which simultaneously sends the input current to the wire under study (R_{wire}) and to a reference resistance (R_{ref}) of identical value as the wire (at low-bias currents), but with a negligible temperature dependence. Since R_{ref} remains unchanged independently of the current, the output signal of the circuit $V_{out} = G(V_{wire}\text{-}V_{ref})$, where G is the gain, simply corresponds to a scaled version of the temperature rise ΔT_{wire} induced in the wire due to Joule heating[2].

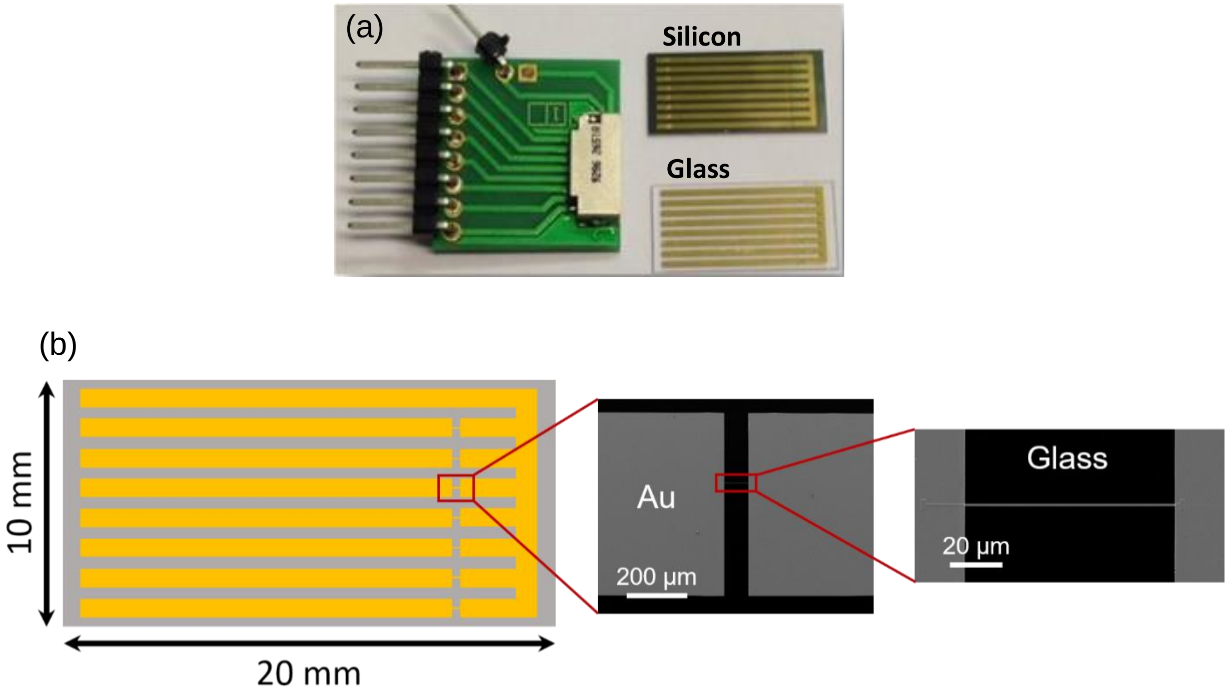

Supplementary Figure 5. Description of the Joule-heated gold nanowires used for nanothermometry. (a) Photograph of the 8-track connector and two chips in silicon and glass. Each chip includes seven nanowires and a common path to connect the circuit to the 8-track connector. **(b)** Schematic representation of a chip and scanning electron microscopy (SEM) images of a 80-µm-long, 1-µm-wide wire on top of a glass substrate.

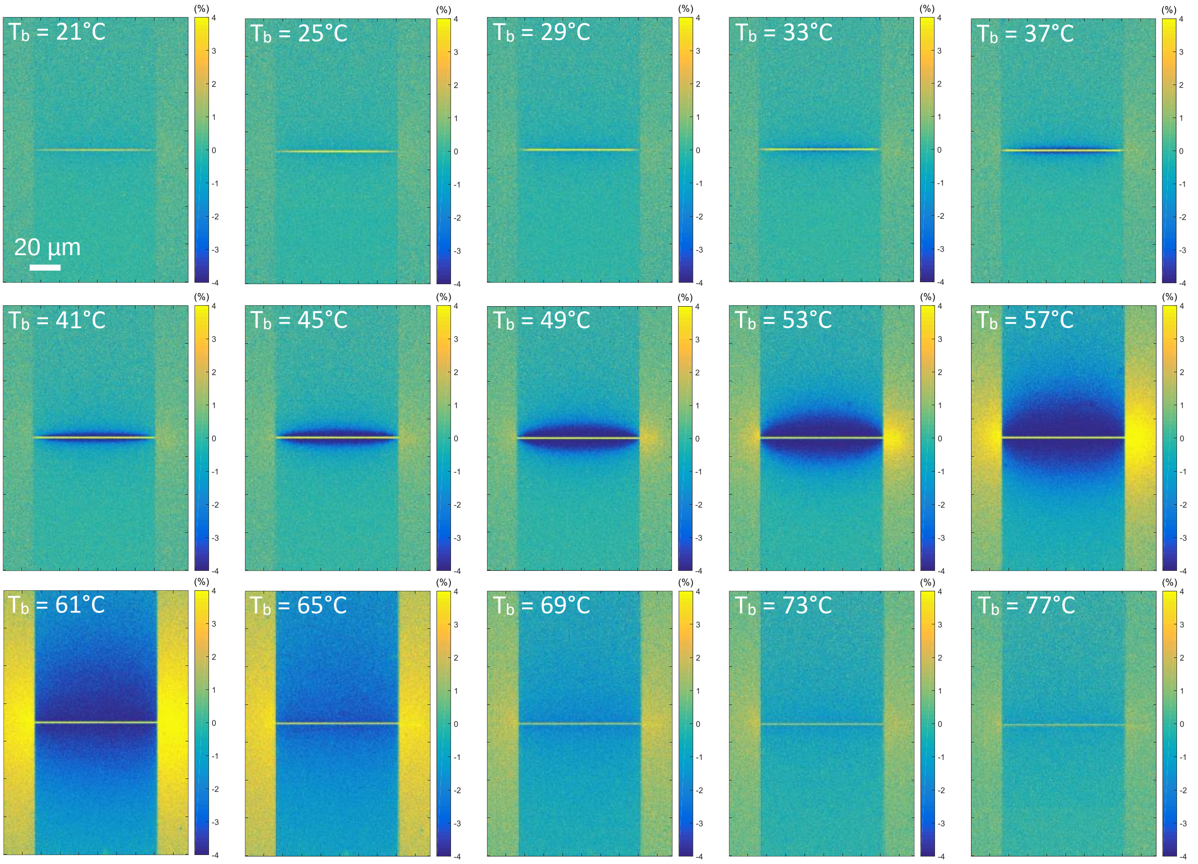

Supplementary Figure 6. Reflectivity images of a Joule-heated nanowire on a glass substrate. Optical reflectivity (λ = 452 nm) images $\Delta R/R = (R_{ON}-R_{OFF})/R_{OFF}$ of a SCO-coated nanowire (80 µm × 1 µm × 50 nm) on top of a glass substrate, recorded at different base temperatures of the substrate ranging from 21 °C to 77 °C. The nanowire is Joule-heated by the application of an electrical current of 4 mA.

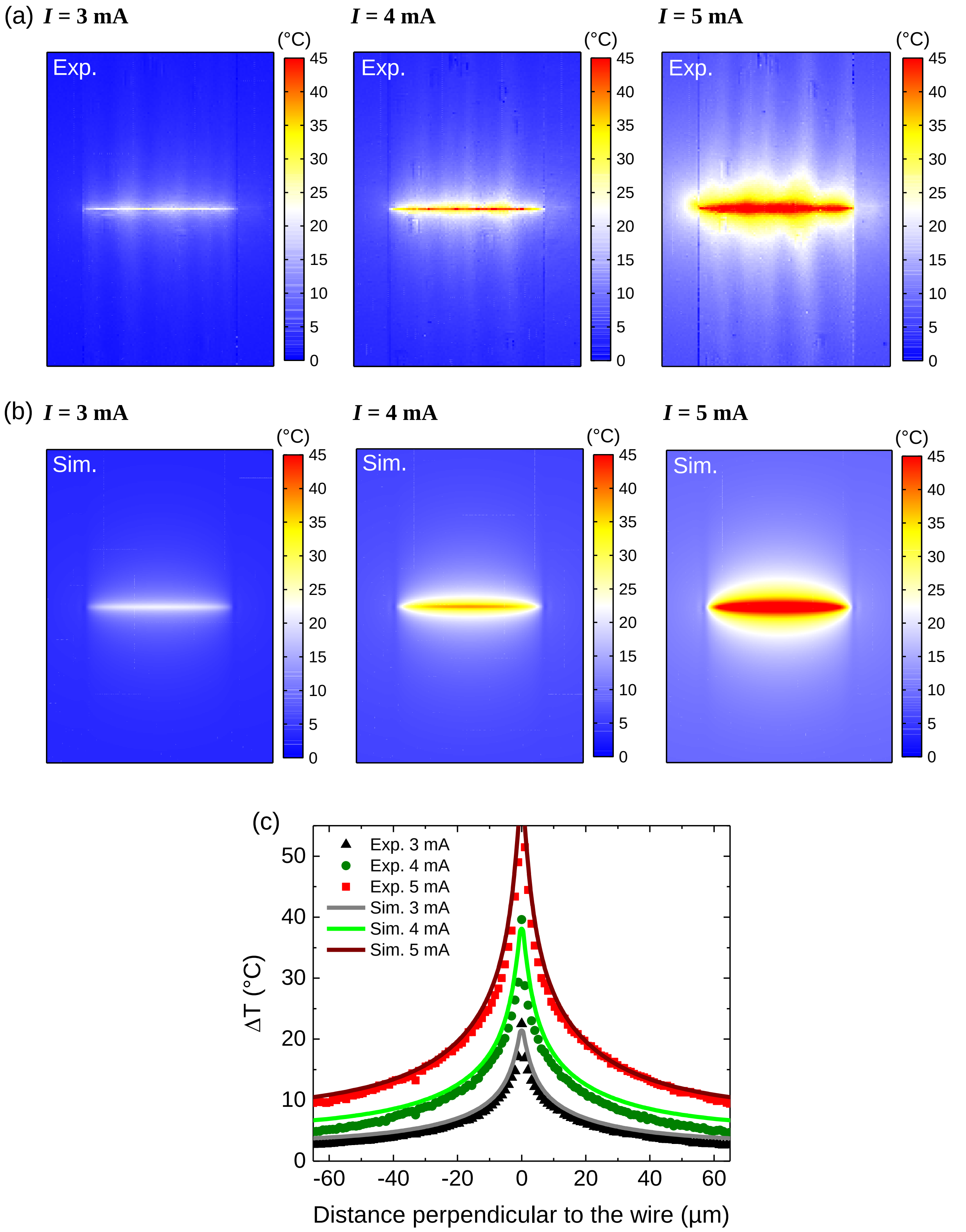

Supplementary Figure 7. Thermal mapping of a Joule-heated gold nanowire on a glass substrate. (a) Experimental and **(b)** simulated temperature maps (ΔT) of a SCO-coated nanowire (80 μm × 1 μm × 50 nm) on top of a glass substrate for different applied electrical currents: 3, 4 and 5 mA. **(c)** Corresponding experimental (data points) and simulated (solid lines) temperature profiles perpendicular to the nanowire.

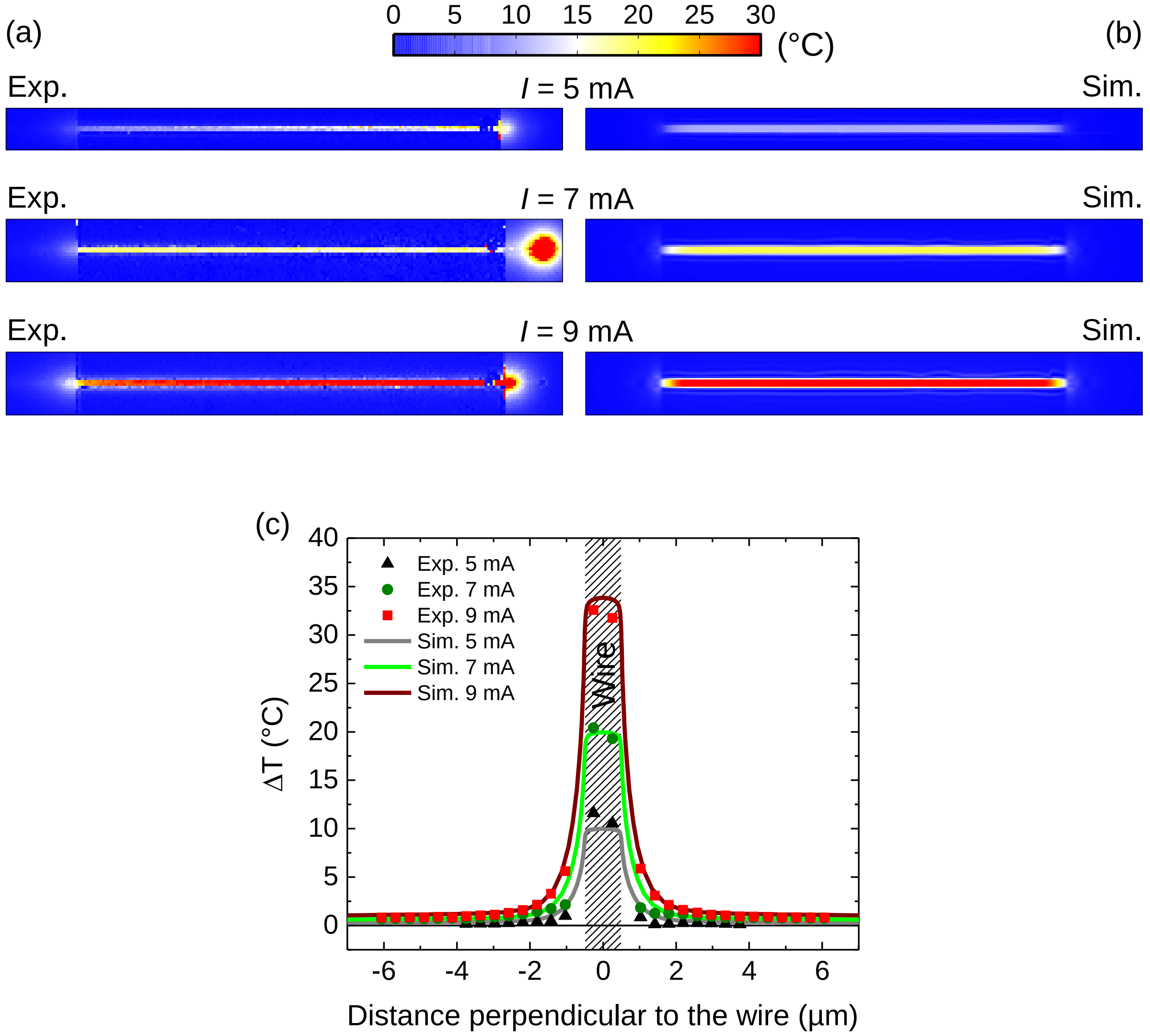

Supplementary Figure 8. Thermal mapping of a Joule-heated gold nanowire on a silicon substrate. (a) Experimental and **(b)** simulated temperature maps (ΔT) of a SCO-coated nanowire (80 µm × 1 µm × 50 nm) on silicon, Joule-heated by the application of various electrical currents: 5, 7 and 9 mA. The measurements were realized in the following chronological order: 5, 9 and 7 mA. A hot spot clearly appears on the temperature map acquired at 7 mA, which is the last measurement performed chronologically, certainly due to a degrading electrical contact at the connection between the wire and the electrode. **(c)** Corresponding experimental (data points) and simulated (solid lines) temperature profiles perpendicular to the nanowire. Contrary to the glass substrate, an abrupt drop of ΔT is observed on Si and the temperature rise completely vanishes at a distance of about 1–2 µm from the center of the wire.

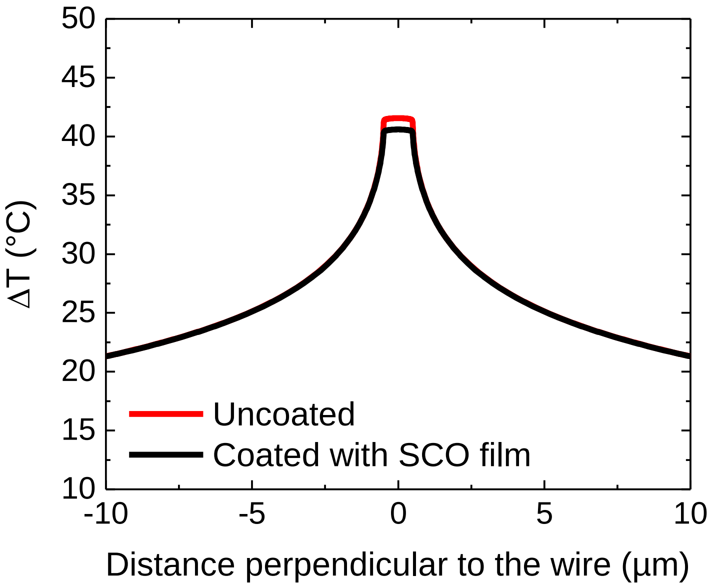

Supplementary Figure 9. Finite-element analysis of the disturbance in the surface temperature distribution induced by the SCO film. Comparative temperature profiles (ΔT), perpendicular to the nanowire on glass, calculated by finite-element simulations, for an uncoated surface and a surface coated with a 200-nm-thick film of **1**. The wire is Joule-heated by the application of an electrical current of 4 mA. In quasi-static conditions, a slight reduction of the temperature (< 1 °C) is observed on the wire due to the presence of the SCO layer. Overall, these simulations show that the disturbance in the heat distribution caused by the SCO thin film remains comparable with the measurement uncertainty. Of course, this effect can be lowered by reducing the film thickness.

(a) T_b = 55 °C (SCO-based thermometry)

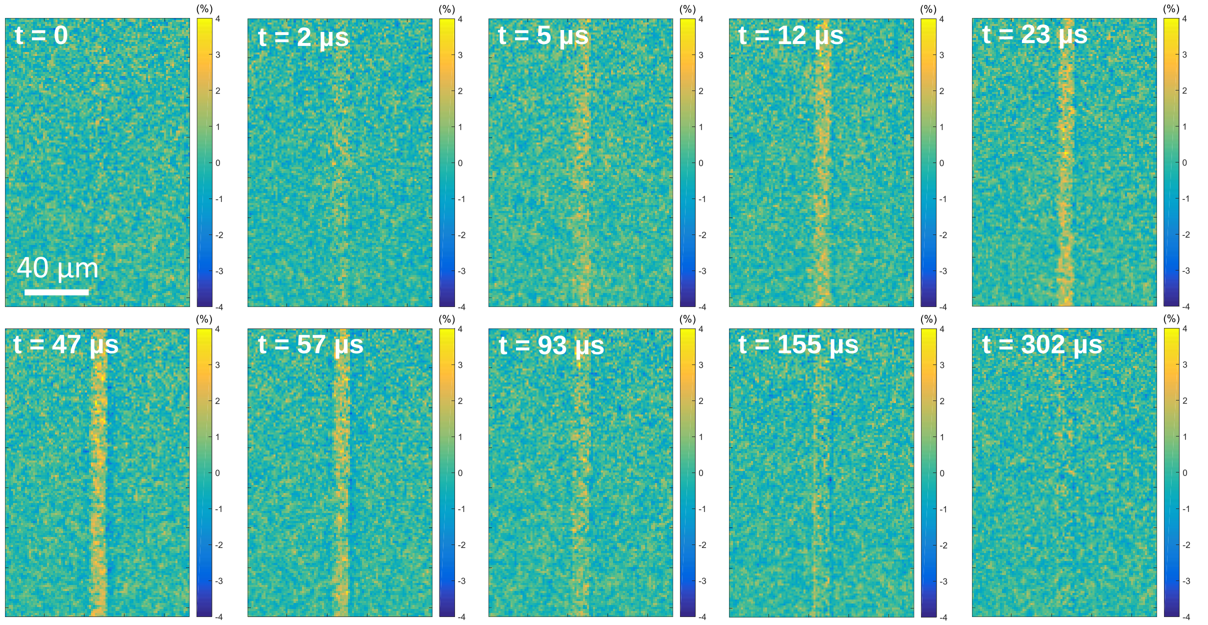

(b) T_b = 25 °C (Conventional thermoreflectance thermometry)

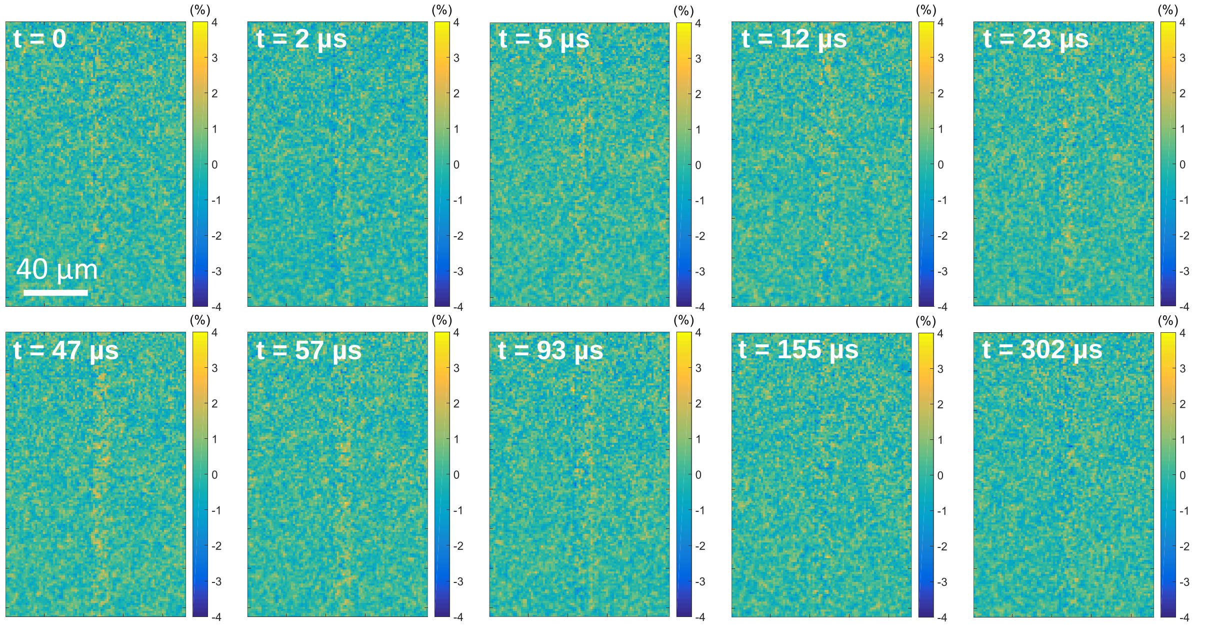

Supplementary Figure 10. Time-resolved measurements. Reflectivity images $\Delta R/R = (R_{ON}-R_{OFF})/R_{OFF}$ of a SCO-coated gold microwire (1 mm × 8 µm × 300 nm) on top of a glass substrate, recorded at selected times following the application of a 50-µs-long current pulse at **(a)** T_b = 55 °C (close to the spin-transition temperature) and **(b)** T_b = 25 °C (far from the spin-transition temperature). The $\Delta R/R$ signal is amplified at 55 °C due to the SCO thin film.

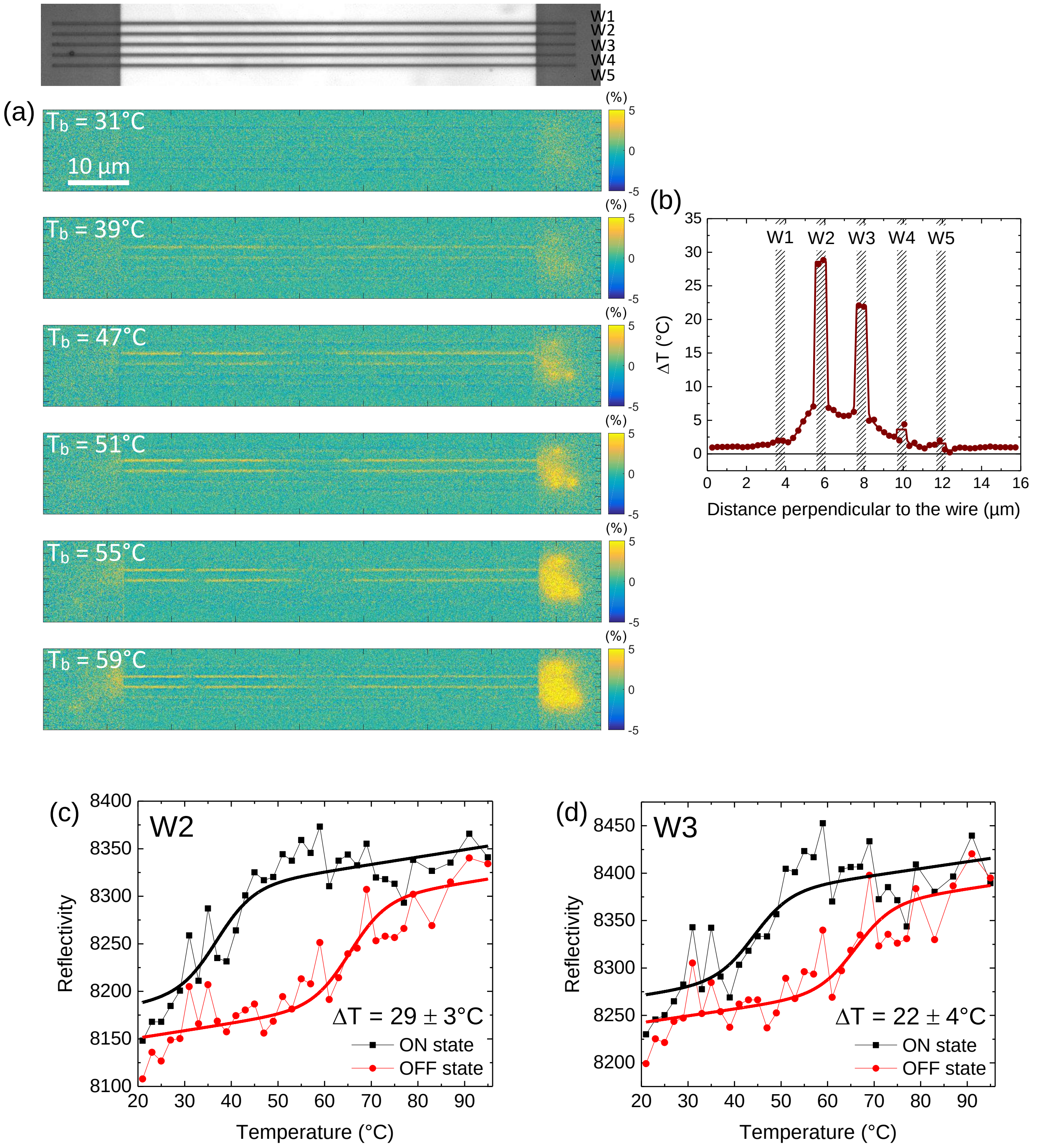

Supplementary Figure 11. Thermal mapping of 500-nm-wide Joule-heated nanowires on a silicon substrate. (a) Reflectivity images $\Delta R/R = (R_{ON}-R_{OFF})/R_{OFF}$ of five 500-nm-wide parallel nanowires on a silicon substrate, numbered from W1 to W5, recorded at different base temperatures of the circuit. The wires are separated by a distance of 1.5 µm and the applied current is 12 mA. Interestingly, a discernable change of the reflectivity is detected only on the wires W2 and W3, while the heating remains almost undetectable on the three other nanowires, revealing a malfunction. **(b)** Temperature profile (ΔT) recorded along the direction perpendicular to the wires. **(c and d)** ON-state and OFF-state reflectivity curves recorded on the whole nanowires W2 and W3. Despite a relatively high noise level of the optical reflectivity signal measured on the wires (because of their small size), an average temperature rise of 29 ± 3 °C and 22 ± 4 °C can be estimated from the curve-fitting procedure for the wires W2 and W3, respectively.

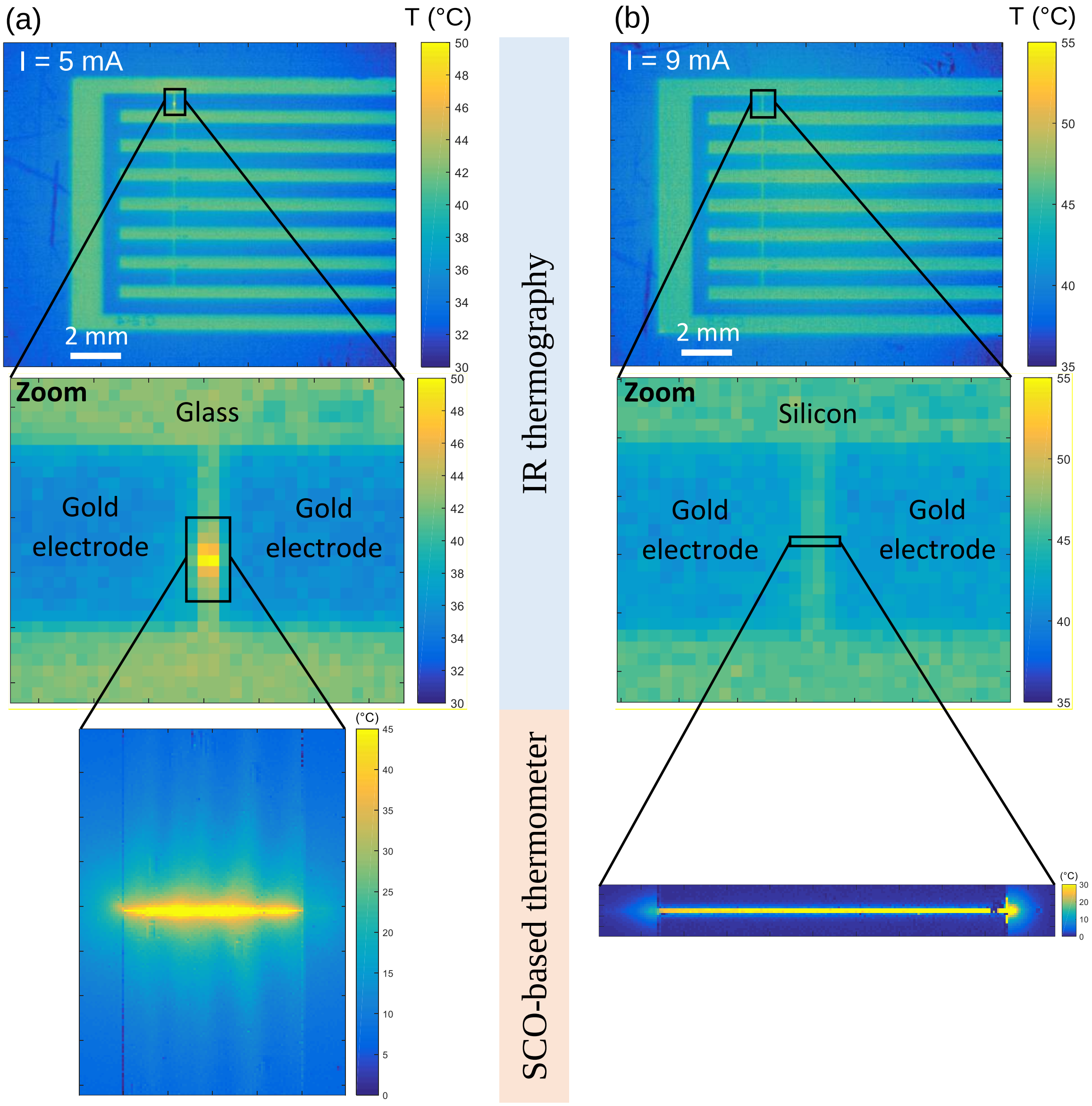

Supplementary Figure 12. Comparison with infrared thermography. Comparative thermal mapping of a Joule-heated gold nanowire (80 µm × 1 µm × 50 nm) on glass and silicon substrates, recorded by infrared thermography (IRT) and by our SCO-based thermometer. **(a)** Thermal images of the nanowire acquired on a glass substrate, Joule-heated by the application of an electrical current of 5 mA. The IRT images were recorded at T_b = 40 °C. **(b)** Thermal images of the same nanowire acquired on a silicon substrate, Joule-heated by the application of a current of 9 mA. The IRT images were recorded at T_b = 45 °C. On the glass substrate (Supplementary Figure 12a), the wire heating is detected by IRT with a very poor accuracy, because the spatial resolution is intrinsically limited by the probe wavelength ($\lambda \sim$ 10 µm) and the instantaneous field of view (28 µm) of the camera used. On the other hand, no sign of heating is detected on the silicon substrate by IRT (Supplementary Figure 12b), because the temperature rise generated by Joule heating is only confined to the 1-µm-wide wire. Note that IR measurements also raise the problem of temperature measurements on heterogeneous surfaces[4], involving different values of emissivity, which does not arise with our thermometry technique.

Supplementary Tables

Supplementary Table 1. Material parameters used in COMSOL Multiphysics simulations (T = 27 °C).

Material	Si	SiO_2	Glass	Ti	Au	SCO
**Thermal conductivity, W.m^{-1}.K^{-1}**	150	1.3	1.3	21.9	317	0.34
**Mass density, kg.m^{-3}**	2330	2200	2210	4506	19300	1530
**Specific heat capacity, J.kg^{-1}.K^{-1}**	700	730	730	522	129	860

Supplementary References

1. Rat, S. *et al.* Solvatomorphism and structural-spin crossover property relationship in bis[hydrotris(1,2,4-triazol-1-yl)borate]iron(II). *CrystEngComm* **19**, 3271–3280 (2017).
2. Quintero, C. M. *et al.* Joule heated metallic microwire devices for sub-microsecond T-jump experiments. *Microelectron. J.* **46**, 1167–1174 (2015).
3. Kraieva, O. *et al.* High Spatial Resolution Imaging of Transient Thermal Events Using Materials with Thermal Memory. *Small* **12**, 6325–6331 (2016).
4. Kim, M. M. *et al.* Microscale thermometry: A review. *Microelectron. Eng.* **148**, 129–142 (2015).
